# Identification of Driver Genes and miRNAs in Ovarian Cancer through an Integrated In-Silico Approach

**DOI:** 10.3390/biology12020192

**Published:** 2023-01-26

**Authors:** Anam Beg, Rafat Parveen, Hassan Fouad, M. E. Yahia, Azza S. Hassanein

**Affiliations:** 1 Department of Computer Science, Jamia Millia Islamia, New Delhi 110025, India; 2 Applied Medical Science Department, CC, King Saud University, Riyadh 11433, Saudi Arabia; 3 Faculty of Engineering and Natural Sciences, International University of Sarajevo, Hrasnička Cesta 15, Ilidža, 71210 Sarajevo, Bosnia and Herzegovina; 4 Biomedical Engineering Department, Faculty of Engineering, Helwan University, Cairo 11792, Egypt

**Keywords:** ovarian cancer, DEMs, miRNA–mRNA network, module

## Abstract

**Simple Summary:**

Ovarian cancer ranks among the most frequent causes of death in women since the prognosis is greatly influenced by the disease’s stage. Since ovarian cancer is largely asymptomatic in its early stages, it is frequently detected in its late stages. One of the hallmarks of ovarian cancer is genomic instability. Although ovarian cancer is divided into several clinical subtypes, each subtype exhibits significant genetic and progressive diversity. In this paper, we concentrate on epithelial ovarian cancer, which is typically discovered when it is already advanced because there is no reliable screening method. Although numerous biomarkers have been researched and used to monitor its status and progression, we still lack drug therapy effectiveness in this subtype. Network biology has recently offered unprecedented opportunities for understanding disease mechanisms from integrative angles. The dysfunctions caused by diseased genes are carried out by a complex network of physical and metabolic interactions. The topological characteristics of these disease genes in the interactome are of particular importance to the systematic comprehension of their activity. We present a systems biology approach to finding miRNAs and complicated disease genes in an integrated biomolecular network in this paper.

**Abstract:**

Ovarian cancer is the eighth-most common cancer in women and has the highest rate of death among all gynecological malignancies in the Western world. Increasing evidence shows that miRNAs are connected to the progression of ovarian cancer. In the current study, we focus on the identification of miRNA and its associated genes that are responsible for the early prognosis of patients with ovarian cancer. The microarray dataset GSE119055 used in this study was retrieved via the publicly available GEO database by NCBI for the analysis of DEGs. The miRNA GSE119055 dataset includes six ovarian carcinoma samples along with three healthy/primary samples. In our study, DEM analysis of ovarian carcinoma and healthy subjects was performed using R Software to transform and normalize all transcriptomic data along with packages from Bioconductor. Results: We identified miRNA and its associated hub genes from the samples of ovarian cancer. We discovered the top five upregulated miRNAs (hsa-miR-130b-3p, hsa-miR-18a-5p, hsa-miR-182-5p, hsa-miR-187-3p, and hsa-miR-378a-3p) and the top five downregulated miRNAs (hsa-miR-501-3p, hsa-miR-4324, hsa-miR-500a-3p, hsa-miR-1271-5p, and hsa-miR-660-5p) from the network and their associated genes, which include seven common genes (SCN2A, BCL2, MAF, ZNF532, CADM1, ELAVL2, and ESRRG) that were considered hub genes for the downregulated network. Similarly, for upregulated miRNAs we found two hub genes (PRKACB and TAOK1).

## 1. Introduction

Ovarian cancer (OC) is among the gynecological malignancies with the highest mortality rates and the lowest cure rates if detected at a late stage. Over a five-year span, the survival rate of ovarian cancer is only 25–30%, with 70–75% of cases being detected at stage III or IV [1]. A silent killer, the symptoms of ovarian cancer are difficult to detect at an early stage. Most patients are diagnosed at the third stage when symptoms start to occur. The reoccurrence rate of ovarian cancer is higher than that of any other gynecological malignancy. Ovarian cancers are classified based on their potential origin in the epithelium, stroma, or germinal cells—the three major components of the ovary. Thus, the primary malignant ovarian tumors are sex cord–stromal tumor, germ cell tumor, and epithelial carcinoma [2]. The most common are epithelial cancers. Eighteen percent of instances of epithelial ovarian cancer, particularly high-grade serous carcinomas, are thought to be caused by inherited genetic defects, mainly BRCA1 and -2 mutations [3]. For decades, combinations of chemotherapies (platinum–taxane) and cytoreductive surgeries have remained the mainstay of therapy. However, the efficacy of a particular treatment in a particular patient cannot be determined by clinicopathological assessments, resulting in excessive treatment plans in many patients with no effective clinical outcome. The development and occurrence of ovarian cancer has been found to be regulated by epigenetic modification in recent studies [4]. Hence, cancer and genetic variations interact, and any variation in the genetic profile can affect disease development and its response to drugs. At present, there is only one clinically approved and implemented biomarker, i.e., CA125, for monitoring ovarian cancer antigens [5]. CA125 expression is increased in the majority of epithelial ovarian malignancies [6]. Nevertheless, for early diagnosis, CA125 cannot be used for prediction as the sensitivity and specificity in early relapse is poor. We therefore need prominent biomarkers that can identify ovarian cancer antigens at an early stage.

The advancement of omics technologies has paved the way for the identification of genes that can differentiate classes of ovarian cancer into molecular subtypes. Research in this field has also found genes that are frequently mutated in ovarian cancer or associated with hereditary ovarian cancer, leading to an enormous amount of data exploring OC resistance, metastasis, development, and progression, and opening frontiers for precision medicine and personalized patient care [4,7,8,9]. In the last few decades, microRNAs (miRNA) have been found to be potent biomarkers in cancer [10,11]. miRNAs modulate gene expression in human ovarian cancers and act as oncogenes, transforming genes, and tumor suppressor genes. Several miRNAs have been identified as potent biomarkers for ovarian cancer survival and response to chemotherapy [12]. However, we still need a full understanding of these miRNAs’ functions in ovarian cancers before clinical trials can be conducted. Hundreds to thousands of genes are targeted by miRNAs, and they may function in organ- or cell-specific manners. Several biological pathways are regulated by a single miRNA that is overexpressed in patients. Since miRNAs meet the criteria of specificity, sensitivity, and accessibility, and they are therefore considered to be ideal biomarkers [13]. miRNAs’ role in ovarian cancer has been investigated in several studies [1,14]. Previous studies have also demonstrated the underexpression of miR-132 in the serum of patients suffering from ovarian cancer [15]. Furthermore, many miRNAs play important roles in early detection [16], overall survival [17], progression-free survival, and resistance to chemotherapy. They have also helped us to further distinguish between disease states [18] after the evaluation of miRNA drug targets were also predicted in various diseases including ovarian cancer [19]. In our study, we identified miRNAs and their associated genes during the expansion and progression of ovarian cancer.

## 2. Materials and Methods

### 2.1. Acquisition of miRNA Expression Data and Ethical Compliance

To analyze the DEGs, we obtained a microarray dataset from the publicly avaiable GEO database by NCBI (https://www.ncbi.nlm.nih.gov/geo/query/acc.cgi?acc=GSE119055, accessed on 14 June 2020). The miRNA dataset GSE119055 includes 6 ovarian carcinoma samples along with 3 healthy/primary samples. The platform used in the GPL21572 dataset is (miRNA-4) Affymetrix Multispecies miRNA-4 Array (ProbeSet ID version, Hunan, China).

The patient data were collected in accordance with GEO’s specifications. Therefore, no consent process or ethics committee approval was required.

### 2.2. Differentially Expressed MicroRNAs (DEMs) Screening

In our study, the DEM analysis of ovarian carcinomas and healthy subjects was conducted using R language version 3.5.0 (https://www.r-project.org/, accessed on 20 June 2020), which was used to transform and normalize all transcriptomic data along with the packages from Bioconductor (http://www.bioconductor.org/, accessed on 9 September 2020). We pre-processed the miRNA expression data by applying the “RMA” algorithm, which involves the adjustment and normalization of the background using the quantile method. The selection of specific DEMs was accomplished using a T static approach with the Linear Models for Microarray “limma” package [20] of Bioconductor. Furthermore, the annotation of DEMs was carried out using the output package, which included an annotation table, and Benjamini–Hochberg (BH) false discovery rate methods were applied to the *p* values. The miRNAs that met the |logFC (fold change)| ≥ 2 for up- and **≤**−2 for downregulated miRNAs and *p*-value < 0.05 primary cut-off criteria were considered DEMs.

### 2.3. Identification of Target Genes of DEMs

Reports over the past five years have shown an uptick in the development of algorithms used to identify the target genes of mammalian genomes’ miRNAs, leading to the creation of 25 miRNA target prediction algorithms [21]. In our study, we performed target prediction for separate upregulated and downregulated miRNAs using three different databases, namely TargetScan, mirDIP, and miRWalk. The TargetScan database uses an algorithm to predict miRNA targets from various genomes and compares multiple genomes to predict targets (http://www.targetscan.org/vert_72/, accessed on 13 July 2020) [22]. miRWalk is a web-based computational method of predicting target sites that is written in the programming language Perl; we used version 3.0 (http://mirwalk.umm.uni-heidelberg.de/, accessed on 12 August 2020) [22,23]. mirDIP is a database that provides extensive, trustworthy, and easy-to-operate resources for miRNA target prediction (http://ophid.utoronto.ca/mirDIP/, accessed on 27 November 2020). Even if one does not have much experience with statistical analysis or computer methods, a wide range of users utilize mirDIP [24]. VENNY 2.1 is the online visualization tool that we used to find common/overlapped genes in all three databases and to categorize them as target/earmarked genes (https://bioinfogp.cnb.csic.es/tools/venny/, accessed on 15 February 2021).

### 2.4. DEM Target Gene Network Construction and Hub Gene Identification

Once the DEMs and target genes have been identified, the process of building networks for DEMs and genes can be started. In this investigation, Cytoscape (Version 3.7.1) was utilized to create a network of DEMs. With the help of Cytoscape and its plugin Cyto-Hubba (version 0.1), various topological techniques, extensive modules, subnetworks, and top-ranking genes/nodes were identified in the network. Cytohubba is used to map the hub genes to their respective miRNAs. For each of the hub genes, miRNAs with a K-core of 2, a degree cutoff of 2, and a node score cutoff of 0.2 were discovered. Finally, using Cytoscape 3.7.1, the hub genes and miRNAs were displayed.

### 2.5. Gene Ontology and Pathway Analysis

After the identification of the DEMs, the gene ontology was analyzed to check if these abnormally expressed genes were linked to any biological/physical processes, molecular/bimolecular functions, or cellular components/functions. This analysis of the gene ontology was conducted using DIANA-mirPath [23]. DIANA mirPath is a web tool for miRNA pathway analysis that also provides KEGG pathway visualization. KEGG (https://www.genome.jp/kegg/, accessed on 1 April 2021) is a collection of databases for deciphering high-level gene functions and biological system utility. An analysis based on pathway enrichment was also carried out using DIANA mirPath.

### 2.6. Transcription Factor and Feed Forward Loop Analysis

TFs are key trans-acting proteins in transcriptional control. Understanding the regulatory circuitry that underpins complex systems is crucial, just as the study of a human illness begins with deciphering the TF–target interactions. To conduct a TF–gene analysis, Enrichr was used, which comprises 35 gene-set libraries, some of which were acquired from other tools and others of which were produced specifically for Enrichr. Enrichr’s gene set libraries are separated into six classes: pathways, ontologies, cell types, transcription, diseases/drugs, and other [25]. Enrichr connects an extensive number of databases. We utilized the TRRUST TF–target interaction database for humans, which was built via sentence-based text mining and was then manually curated [26]. miRNAs and TFs are the two key factors for biological process, viz. transcriptional/post-transcriptional control. It is crucial to understand how the two regulators interact with their targets to decipher complicated molecular regulatory systems. FFLtool, a web tool for detecting the possible FFL of TF–miRNA–target regulation linked to ovarian cancer, was employed in this study [27].

## 3. Results

### 3.1. Identification of DEMs Associated with Ovarian Cancer

**Figure 1:**
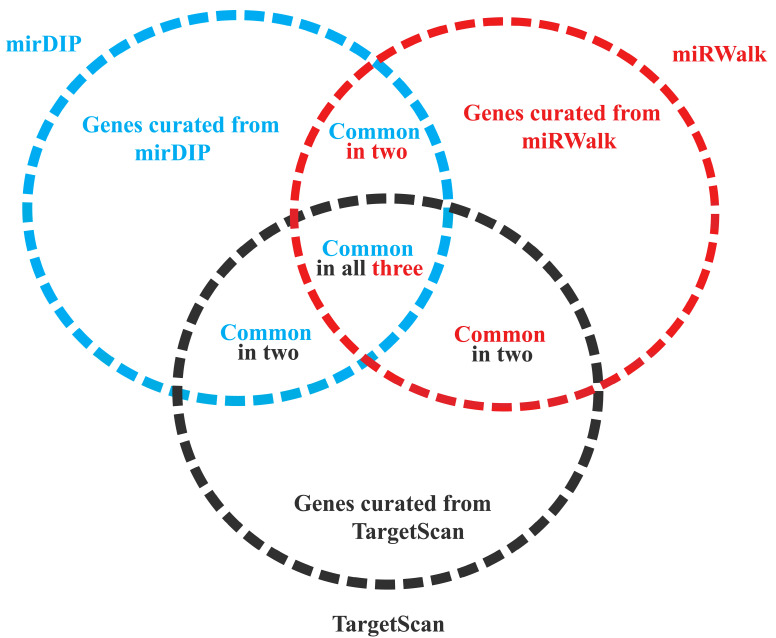
The overlapping genes taken from all three databases were found using a Venn analysis.

For the identification of anomalies in miRNA involved in ovarian cancer, GEO dataset GSE119055 is considered. This retrieved dataset includes a total of nine samples, including six ovarian carcinoma samples and three healthy samples. A total of 4575 miRNAs were found in GSE119055, of which 42 DEMs were distinguished. Total miRNAs from the series and final upregulated and downregulated miRNAs on the basis of the *p* value and log Fc can be found in the supplementary material S1. Other statistical parameters were also applied and are given in Table 1. Based on these parameters, 15 upregulated and 27 downregulated miRNAs were extracted. The expression levels of the top five upregulated and downregulated miRNAs in the dataset are listed in Table 1. miRNAs regulate target genes by binding to their complementary sites, resulting in the silencing of their post-transcriptional modifications. To extensively study the probable function of the top DEMs in ovarian cancer, we placed 741 overlapped genes/targets of the top five downregulated miRNAs and 1115 overlapped genes/targets of the top five upregulated miRNAs in a Venn diagram using VENNY 2.1 (Figure 1). These target genes were found in three different databases, including TargetScan, mirDIP, and miRWalk. Common genes sorted from all the three databases were given in Table S2.

Top 10 DEMs were extracted from These 741 downregulated genes and 1115 upregulated. For each downregulated miRNA, the number of target genes was: hsa-miR-501-3p = 118; hsa-miR-4324 = 86; hsa-miR-500a-3p = 22; hsa-miR-1271-5p = 417; and hsa-miR-660-5p = 98. For the upregulated miRNAs: hsa-miR-130b-3p = 106; hsa-miR-18a-5p = 220; hsa-miR-182-5p = 724; hsa-miR-187-3p = 14; and hsa-miR-378a-3p = 51. 

### 3.2. DEM–miRNA Network Construction and Extraction of Disease-Associated Genes

**Figure 2:**
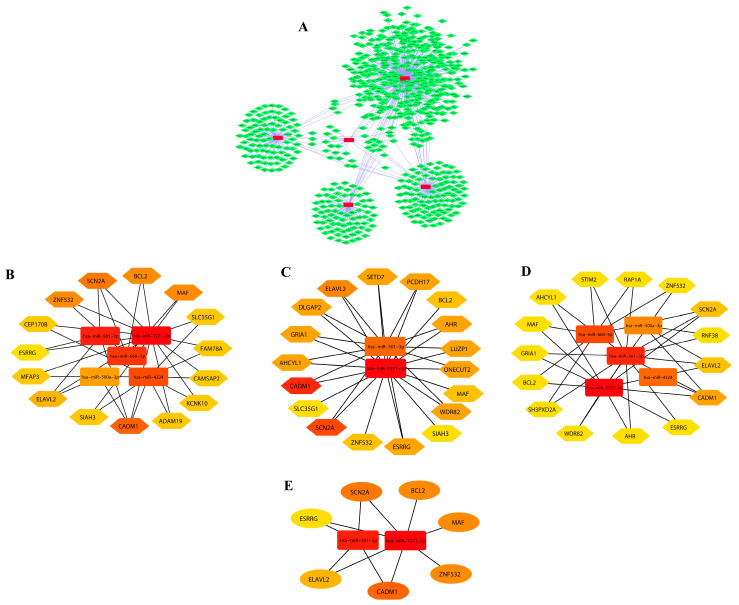
(**A**) Downregulated network of miRNAs and their associated target genes. The associated genes were further identified on the basis of centrality measures. (**B**) On the basis of betweenness. (**C**) On the basis of closeness. (**D**) On the basis of degree. (**E**) Common genes obtained from significant modules.

**Figure 3:**
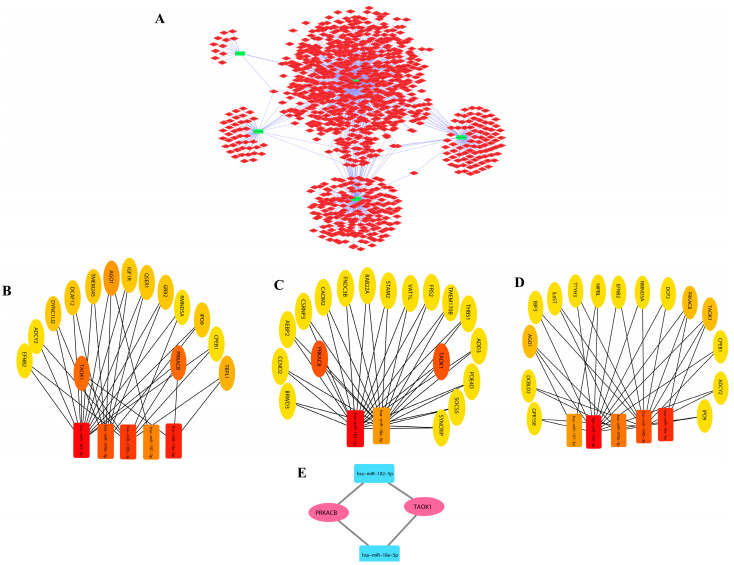
(**A**) Upregulated network of miRNAs and their associated target genes. The associated genes were further identified on the basis of centrality measures. (**B**) On the basis of betweenness. (**C**) On the basis of closeness. (**D**) On the basis of degree. (**E**) Common genes obtained from significant modules.

A regulatory network of miRNAs and their target genes was constructed for both upregulated and downregulated miRNAs using cytoscape (version3.6.1). It was discovered that the top five (hsa-miR-501-3p, hsa-miR-4324, hsa-miR-500a-3p, hsa-miR-1271-5p, and hsa-miR-660-5p) downregulated miRNAs regulate around 701 disease-associated genes (Figure 2A) in the network, and there are 740 edges that show interconnections between the miRNAs and genes. Similarly, the top five (hsa-miR-130b-3p, hsa-miR-18a-5p, hsa-miR-182-5p, hsa-miR-187-3p, and hsa-miR-378a-3p) upregulated miRNAs regulate around 1048 disease-associated genes (Figure 3A) in the network, and there are 1114 edges that show interactions between the miRNAs and genes. The network images of downregulated and upregulated miRNAs are represented below.

### 3.3. Module Detection and Pathway Enrichment Analysis

The miRNAs and genes involved in ovarian cancer obtained from both the upregulated and downregulated networks were shortlisted on the basis of centrality metrics to lessen complications and the intervention of unconnected genes. Hence, for the network of downregulated miRNAs and their associated target genes, 20 nodes and 31 edges based on betweenness metrics, 20 nodes and 34 edges based on closeness metrics, and 20 nodes (gene/miR) and 34 edges based on degree metrics were obtained (Figure 2B–D, respectively). A total of seven common genes (SCN2A, BCL2, MAF, ZNF532, CADM1, ELAVL2, and ESRRG) were selected as hub genes (Figure 2E) obtained from notable modules of betweenness, closeness, and degree.

Similarly, for the network of upregulated miRNAs and their associated target genes, we obtained 20 nodes and 36 edges based on betweenness metrics, 20 nodes and 33 edges based on closeness metrics, and 20 nodes (gene/miR) and 33 edges based on degree metrics (Figure 3B–D, respectively). A total of two common genes (PRKACB and TAOK1) were selected as hub genes (Figure 3E).

**Figure 4:**
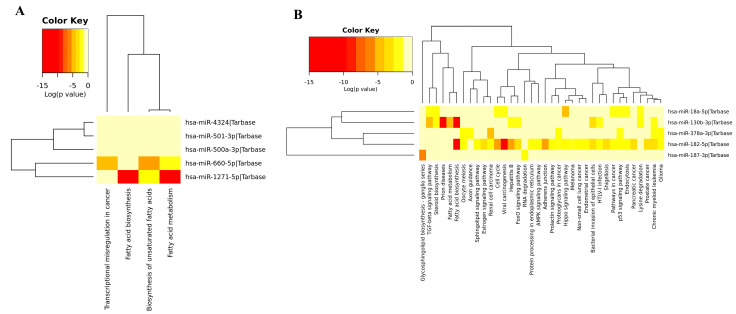
KEGG pathway enrichment analysis. (**A**) Downregulated miRNAs. (**B**) Upregulated miRNAs.

The color in the modules shows the rank of the first 20 nodes. The top and bottom ranks are represented by dark and light colors, respectively. We used online databases including TRRUST and Enrichr to uncover transcription factors (TFs) for these hub genes. These hub genes were also utilized to locate interacting partners in the overall network. These hub genes, on the other hand, were linked to miRNAs (Figure 2A and Figure 3A). Furthermore, we used Cytoscape (version 0.1) with the plugin CytoHubba to conduct a network analysis with a score approach. DIANA-mirPath was used to carry out a pathway enrichment analysis of the DEMs, which included KEGG. A heat map of these DEMs (up and down) was created using the DIANA-mirPath v3.0 interface to display the route enrichment analysis results (Figure 4).

### 3.4. Gene Ontology of DEM

**Figure 5:**
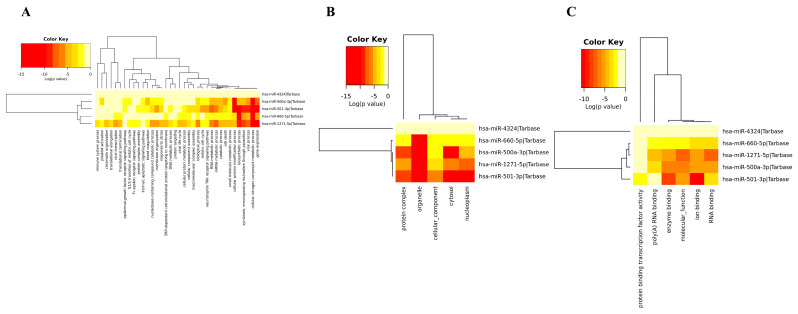
Gene ontology of downregulated miRNAs. (**A**) Biological processes. (**B**) Cellular components. (**C**) Molecular functions.

**Figure 6:**
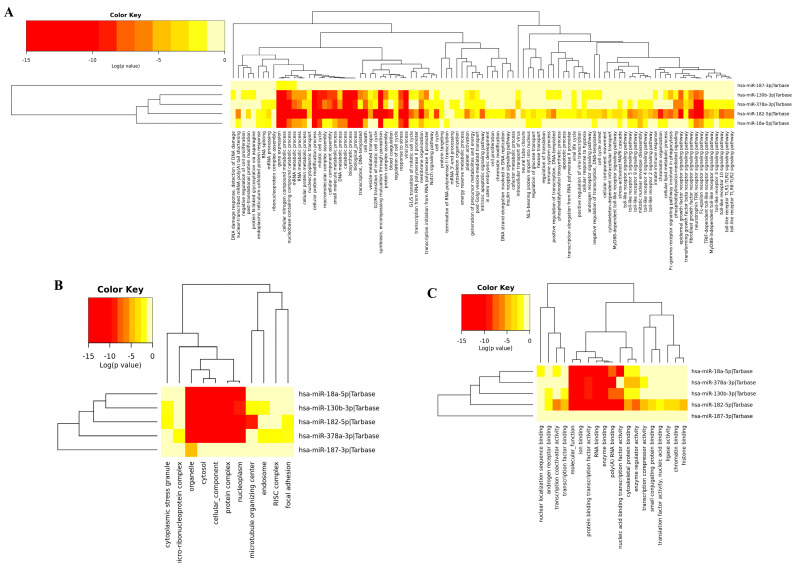
Gene ontology of upregulated miRNAs. (**A**) Biological processes. (**B**) Cellular components. (**C**) Molecular functions.

The functional enrichment of the top downregulated and upregulated DEMs (Figure 5 and Figure 6) were analyzed using the DIANA-mirPathV3 database (http://amp.pharm.mssm.edu/Enrichr/, accessed on 9 September 2021). To find the gene ontology functional annotation, DIANA-mirPath was used with <0.05 being considered a significant statistical *p* value. All the DEMs in both the upregulated and downregulated networks possess some cellular biological processes that involve protein modification, nitrogen metabolism, and biosynthetic processes. Thus, we performed an analysis of the gene sets generated from the GWAS experiment. Our results show which function, component, and biological processes the DEMs with enriched molecular functions are connected with.

### 3.5. Transcription Factor Finding and Feed Forward Loop Analysis

Different cellular responses and developmental fates that lead to environmental and genetic changes are controlled by transcriptional regulation. Transcription factors are among the most important trans-acting proteins because they bind to cis-regulatory regions of DNA to activate RNA polymerase and initiate the transcription of targeted genes. TF mutations typically result in the dysregulation of target and downstream genes, and differentiation can be induced by different TFs in different cell types [26].

Following the preparation of an unbiased number of genes from such a study, they are used as inputs for in silico enrichment using previously generated lists organized into gene-set libraries. These repositories are used to store and manage information on the function of groups of genes. Each gene-set library is composed of a collection of relevant gene lists that are connected to a functional term, such as a pathway or a transcription factor that regulates genes. TRRUST was used in this work alongside Enrichr, a web-based integrative server with multiple new gene-set libraries, a unique approach to rating enriched words, and a sophisticated interactive method of visualizing data in new ways [25]. Enrichr is a tool for detecting TF–target gene interactions.

**Figure 7:**
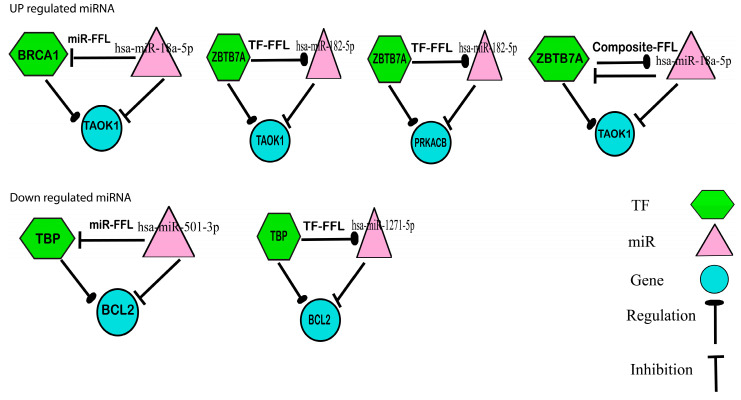
The biological connectivity between genes, miRNAs, and transcription factors.

TBP–BCL2, BRCA1–TAOK1, ZBTB7A–TAOK1, and ZBTB7A–PRKACB interactions were consequently discovered. TFs and miRNAs are two types of key regulators involved in transcriptional and post-transcriptional modifications. It is crucial to understand how the two regulators interact with their targets in order to decipher their complicated molecular regulatory systems. FFLtool is a web tool that was employed in this study to decipher the possible feed forward loop of TF–miRNA–target regulation in humans. We incorporated extensive TF–target and miRNA–target regulations into FFLtool and built two different functional modules: (i) The “FFL Analysis” module may identify plausible FFLs and internal regulatory networks in a user-defined gene collection. (ii) The “Browse FFLs” module reveals FFLs made up of differentially or especially expressed TFs and miRNAs, as well as their target genes in carcinoma, with three levels of evidence indicating the reliability of each FFL and enrichment functions for co-target genes of the same TF and miRNA. The FFLtool is a helpful tool for understanding gene expression control and mechanisms in biological processes and diseases [27]. Figure 7 depicts the biological connectivity between genes, miRNAs, and transcription factors.

## 4. Discussion

In this study, bioinformatics and an in-silico approach were used on the microarray dataset GSE119055 of ovarian cancer to screen miRNAs and their associated genes that may be responsible for the prognosis of OC patients. In the present study, nine samples were included from the retrieved dataset of GSE119055, incluing six ovarian carcinoma samples and three healthy samples. In the GSE119055 series, a total of 4575 miRNAs were found. Based on a gene expression analysis, 42 DEMs were obtained that met two thresholds, i.e., *p* value and log Fc (previously explained) value, to extract upregulated and downregulated miRNAs. Based on these parameters, we extracted 15 upregulated and 27 downregulated miRNAs. The expression levels of the top five upregulated miRNAs (hsa-miR-130b-3p, hsa-miR-18a-5p, hsa-miR-182-5p, hsa-miR-187-3p, and hsa-miR-378a-3p) and the top five downregulated miRNAs (hsa-miR-501-3p, hsa-miR-4324, hsa-miR-500a-3p, hsa-miR-1271-5p, and hsa-miR-660-5p) is described in Table 1. Of these 10 miRNAs, hsa-miR-660-5p is important because numerous studies have linked hsa-miR-600 to a number of cancers, including ovarian cancer [28], cervical cancer [29], colorectal cancer [30,31], breast cancer [32], and even small cell lung carcinoma [33]. Additionally, it has been found that 5p is linked with various miRNAs and is associated with numerous diseases, including cancer [34,35,36,37]. As a result, it is hypothesized that if hsa-miR-660 plays a significant role in these cancers and 5p is also linked with different diseases, then hsa-miR-660-5p might be connected with the illness.

We further analyzed the data set GSE131790 to validate a few results using GEO2R. The LIMMA package was used, and we found that the hsa-miR-501-3p miRNA had a significant *p*-value (7.28 × 10^−9^) and logFC (−3.38967), which show downregulation. This verifies that hsa-miR-501-3p miRNA was downregulated in this study.

Additionally, the study’s methodology has undergone validation. Numerous investigations have revealed that the miRNA hsa-miR-130b-3p, which was discovered in this study, is linked to the upregulation of epithelial ovarian cancer [38] and that hsa-miR-18a-5p is linked to ovarian cell proliferation [39] and various other diseases [40]. It is also linked to the upregulation of epidermal necrolysis in severe drug eruptions [41]. In this research, we found that it was upregulated.

The current research findings that hsa-miR-182-5p is elevated and hsa-miR-1271-5p is downregulated in human epithelial ovarian cancer were confirmed by previous studies [42]. Similarly, another miRNA hsa-miR-378a-3p that is upregulated in this research was also found to be upregulated in squamous cell carcinoma [43] and various other diseases [44].

Hence, hsa-miR-501-3p [45], hsa-miR-1271-5p [42], and miR-18a-5p [39] play important roles in ovarian cancer, and the miRNAs hsa-miR-4324, hsa-miR-500a-3p [46], and hsa-miR-187-3p play crucial roles in various cancers, but their role in ovarian cancer is yet to be discovered [46,47,48,49]. Since miRNA binds to the complementary sites of target genes, it exerts its regulatory function on them, so we need to know the associated target genes of these miRNAs. With the help of different databases (Target Scan, miRDIP, and miRWALK), target genes were retrieved, and with the Venny tool we found common targeted genes in all three databases.

Hence, to identify the potential role of the top DEMs, we obtained 741 target genes of the top five downregulated miRNAs and 1115 target genes of the top five upregulated miRNAs. Further, we found that of those 741 and 1115 targeted genes, 701 and 1048 were disease-associated genes. Therefore, to lessen the complications and involvement, these genes were separated from the list of ovarian cancer-associated genes in both the upregulated and downregulated networks. Based on centrality, the top 20 were identified. For the network of downregulated miRNA, seven common genes were identified (SCN2A, BCL2, MAF, ZNF532, CADM1, ELAVL2, and ESRRG) that were considered hub genes acquired from significant modules based on the parameters of betweenness, closeness, and degree. Similarly, for upregulated miRNAs, two hub genes were found (PRKACB and TAOK1). All the genes found are significant and play important roles in ovarian cancer and various other cancers, but the role of ZNF532 in ovarian cancer is yet to be discovered. Additionally, this research provides the first in silico study on ESRRG’s involvement in ovarian cancer. It has been studied in pervious research [50,51]. The details of each gene are provided in Table 2. Further, Enrichr was used to detect interactions between TFs and target genes. TBP–BCL2, BRCA1–TAOK1, ZBTB7A–TAOK1, and ZBTB7A–PRKACB interactions were discovered to deepen our understanding of genes’ mechanisms, functions, and involvement.

## 5. Conclusions

Although significant advancements have been made in the past 50 years, the early diagnosis of ovarian cancer is still a challenge, and therefore a computational system-based method offers a systematic framework for establishing a link between biomarker candidates and driving functional dependencies between clinically interconnected disorders. With the massive volume of data generated from current cancer clinical investigations, a standardized data analysis process is essential to assist researchers in retrieving prospective biomarkers from data. This research establishes that miRNAs have a broad impact on gene expression patterns in ovarian cancer, adding to our understanding of miRNA biology in human cancer. miRNAs, like gene transcripts, have a lot of variation, which reflects the genetic variability within a clinically homogeneous illness population. Overall, our findings may provide useful guidance for biomarker research into ovarian cancer prognosis. Based on a microarray analysis, critical miRNAs and their associated genes were identified, and their biological involvement was confirmed using GSE119055 data. Ten miRNAs were discovered that might show a high level of diagnostic and prognostic accuracy in ovarian cancer. These hub genes will be further screened for survival analysis, mutational analysis, and therapeutic target selection against selected genes. Hence, these results can be further utilized for the selection of drug targets helpful for diagnosis and the development of drugs. Since this was an in silico study, clinical and experimental validation is required to confirm all conclusions regarding miRNAs and their associated genes.

## Figures and Tables

**Table 1:** The top five upregulated and top five downregulated miRNAs based on the logFc and *p*-value.

ID	miRNA ID	Log FC	*p*-Value
**Up-regulated**			
20501181	hsa-miR-130b-3p	2.00379	0.000894
20500132	hsa-miR-18a-5p	2.172073	0.006009
20500450	hsa-miR-182-5p	3.910191	0.006054
20500455	hsa-miR-187-3p	2.440276	0.008152
20501243	hsa-miR-378a-3p	2.4392	0.017887
**Down-regulated**			
20503878	hsa-miR-501-3p	−2.589788641	2.21 × 10^−6^
20517704	hsa-miR-4324	−2.773375005	3.00 × 10^−5^
20503876	hsa-miR-500a-3p	−2.197486478	8.40 × 10^−5^
20504569	hsa-miR-1271-5p	−2.517825869	0.00020669
20504431	hsa-miR-660-5p	−2.392424335	0.00025731

**Table 2:** Brief description of all the discovered genes based on chromosomal location, aliases, functions, expression, and gene structure.

	Genes	Chromosomal Location	Aliases	Expression	Gene Structure	Functions	Reference
Downregulated Genes	**SCN2A** (sodium voltage-gated channel alpha subunit 2)	2q24.3	EA9; HBA; NAC2; BFIC3; BFIS3; Nav1.2; SCN2A1;	Biased expression in kidney and brain	120kb in size and have 29 exons	Enables protein binding and voltage gated sodium channel activity	[52]
	**BCL2** (Apoptosis regulator)	*20q11.21* 18q21.33	Bcl-2; PPP1R50	Broad expression is found in thyroid, spleen, and 20 different tissues such as ovary, prostate, colon, skin endometrium placenta, lungs, etc.	6exons	Enables channel activity, protease binding, protein binding, etc.	[53]
	**MAF** (MAF bZIPtranscription factor)	*16q23.2*	CCA4; AYGRP; c-MAF; CTRCT21	Ubiquitous expression in kidney, endometrium, and 24 other tissues	7 exons	Role in RNA polymerase II-specific oncogenesis activator activity	[54]
	**ZNF532** (zinc finger protein 532)	18q21.32	-	Ubiquitous expression in endometrium, prostate, and 22 other tissues	21 exons	Enables DNA binding and metal ion binding, and along with ncRNAs it is involved in apoptosis	[55]
	**CADM1**	11q23.3	BL2; ST17; IGSF4; NECL2; RA175; TSLC1;	Broad expression in lung, thyroid, and 23 other tissues	13 exons	Enables signaling receptor binding, PDZ domain binding, and protein binding	[56,57]
	**ELAVL2**	9p21.3	HUB; HELN1; HEL-N1	Biased expression in testis and brain	20 exons	Enables RNA binding, protein binding, and activation of protooncogenes	[58]
	**ESRRG**	1q41	ERR3; ERRg; NR3B3; ERRgamma; ERR-gamma	Biased expression in kidney, stomach, and 12 other tissues	28exons	Enables nuclear receptor activity and protein binding	[59]
Upregulated Genes	**PRKACB** (protein kinase cAMP-activated catalytic subunit beta)	1p31.1	CAFD2; PKACB; PKA C-beta	Broad expression in brain, colon, and 22 other tissues	18 exons	Enables ATP binding and caMP dependent protein kinase activity	[60]
	**TAOK1** TAO kinase 1	17q11.2	PSK2; TAO1; KFC-B; MARKK; PSK-2; MAP3K16	Ubiquitous expression in brain, thyroid, and 25 other tissues	20 exons	Enables ATP binding, kinase activity, and protein binding	[61,62,63])

## Data Availability

The dataset used in the current study is publicly available at the NCBI GEO dataset for ovarian cancer. The persistent link can be found at: https://www.ncbi.nlm.nih.gov/geo/query/acc.cgi?acc=GSE119055.

## Supplementary Materials

The following supporting information can be downloaded at: https://www.mdpi.com/article/10.3390/biology12020192/s1, Table S1: Total miRNAs from the series and final upregulated and downregulated miRNAs on the basis of the *p* value and log Fc; Table S2: Common genes sorted from all the three databases.

## Abbreviations

OC	Ovarian Cancer
GEO	Gene Expression Omnibus
miRNA	Micro RNA
DEMs	Differentially Expressed miRNAs
DEGs	Differentially Expressed Genes
KEGG	Kyoto Encyclopedia of Genes and Genomes
TFs	Transcription Factor
TRRUST	Transcriptional Regulatory Relationships Unraveled by Sentence based Text mining
FFL Tool	Feed Forward loop
